# COVID-19: the case for aerosol transmission

**DOI:** 10.1098/rsfs.2021.0072

**Published:** 2022-02-11

**Authors:** Raymond Tellier

**Affiliations:** Department of Medicine, McGill University, Montreal, Quebec, Canada

**Keywords:** COVID-19, aerosol, transmission

## Abstract

The COVID-19 pandemic is the most severe pandemic caused by a respiratory virus since the 1918 influenza pandemic. As is the case with other respiratory viruses, three modes of transmission have been invoked: contact (direct and through fomites), large droplets and aerosols. This narrative review makes the case that aerosol transmission is an important mode for COVID-19, through reviewing studies about bioaerosol physiology, detection of infectious SARS-CoV-2 in exhaled bioaerosols, prolonged SARS-CoV-2 infectivity persistence in aerosols created in the laboratory, detection of SARS-CoV-2 in air samples, investigation of outbreaks with manifest involvement of aerosols, and animal model experiments. SARS-CoV-2 joins influenza A virus as a virus with proven pandemic capacity that can be spread by the aerosol route. This has profound implications for the control of the current pandemic and for future pandemic preparedness.

## 1. Introduction

As this review is being written, the COVID-19 pandemic is still on rampage on a global scale, in spite of the increasing availability of effective vaccines. The rise of the new delta variant [1], significantly more contagious than the original lineage, contributes to the disease outpacing efforts to slow the spread of SARS-Cov-2. It is likely that non-pharmaceutical interventions will have to continue to play a role in mitigating outbreaks while vaccination catches up.

How SARS-CoV-2 spreads, then, matters for a rational approach to interrupt the chains of transmission. For respiratory viruses, three routes have been implicated: contact with virus-containing secretions (either directly or through fomites); ‘large droplets’, falling quickly to the ground along a ballistic trajectory within a short radius; and aerosol transmission. Aerosol transmission is the most controversial route, for a variety of reasons, including difficulties in its demonstration and reluctance to commit to the significant efforts required to block this form of transmission, which require specialized personal protective equipment (PPE) and environmental controls such as ventilation and air filtration. Of course, these three modes of transmission are not mutually exclusive.

## 2. SARS-CoV-2 and some fundamental concepts of aerobiology

Aerosols are defined as suspensions of particles in a gas, e.g. in air; small particles in still air will settle with a velocity that depends on their aerodynamic diameter and which can be calculated to a good precision using Stokes’ Law [2]. Suspension in air can be more prolonged if the air is stirred; an important property of aerosols is that they will follow air jets or currents. These properties vary continuously with the aerodynamic diameter, making difficult the definition of a size cut-off for aerosols. This is an issue relevant to bioaerosols since humans produce through coughing and sneezing a large size spectrum of droplets from the sub-micrometre to several hundreds of micrometres (reviewed in [3]). Traditionally the proposed size cut-off for ‘bioaerosols’ was less than 5–10 µm; but it is now appreciated that particles much larger that this will in fact be carried away by air currents or air jets well beyond the canonical 2 m distance [4,5]; furthermore, coughing, or even exhalation, injects a hot, humid air jet into cooler, drier air; humidity and heat have a buoyancy effect that will keep small particles suspended longer and carried away further, especially for particles less than 10 µm [6,7].

Currently, a new consensus is emerging that bioaerosols up to 100 µm would count as aerosols [4,8,9]. Admittedly, however, an emphasis on the smaller end of the spectrum (‘fine’ aerosols) is often appropriate or interesting since they not only remain suspended longer but can penetrate deeper in the human respiratory tract; significant penetration below the glottis occurs at diameters less than 20 µm, and into the alveolar space at less than 5–6 µm [2]. This is immediately relevant to infectious agents causing infections restricted to the lower respiratory tract, for example, tuberculosis or MERS-CoV [4,10].

*Droplet nuclei* arise through the phenomenon of desiccation of bioaerosols, where water loss occurs by evaporation while non-volatile solutes such as salts, proteins, etc., are retained [3]. At a relative humidity (RH) of 50%, bioaerosols will shrink to approximately half their initial diameter [3]. This process occurs rapidly: for bioaerosols with an initial diameter of less than 20 µm, desiccation at a RH of 50% occurs in less than 1 s, and for particles of less than 50 µm in less than 4 s [3,11]. As infectivity of aerosolized viruses persist for hours, the dessication can be treated as occurring instantaneously and the concept of droplet nuclei is arguably not very useful in practice, except for an interesting aspect of pathogenesis involving the difference between penetration and deposition of aerosols in the respiratory tract. Fine aerosols can penetrate all the way into the alveolar space, but only a fraction will be deposited, the rest is exhaled back (but can be inhaled again) [12]. Droplet nuclei are hygroscopic: when exposed again to air at a RH of 100% (as in the alveolar space) they will swell back to their original size and so one would expect that a higher fraction would be deposited, compared to non-hygroscopic particles: this has been verified experimentally [13].

Infectious aerosols can in some circumstances cause long-range transmission; when this is observed it essentially constitutes a proof of aerosol transmission. However, long-range transmission is modulated by several factors such as removal by ventilation/filtration, biological decay, the magnitude of the infectious dose, etc.; and so the lack of observed long-range transmission does not rule out a role for aerosols. Indeed aerosols will be present at higher concentration near the source, and mathematical modelling shows that at close range aerosol transmission would even predominate over large droplets unless the distance is less than 0.5–0.6 m [14,15].

Detection of viruses of interest in aerosol samples collected from the air can be readily achieved by molecular methods. Detection of *infectious* viruses from these samples by isolation in cell culture is, however, significantly less sensitive for two reasons: firstly the infectious dose for cell culture typically consists of at least several hundreds of virions, and secondly several classical methods of aerosol sampling damage the virions and abrogate their infectivity. Consequently, failure to isolate viruses in cell culture from an aerosol sample does not completely rule out the presence of infectious viruses in aerosols, and especially if the sample contains a low viral load. Measurements of the ratio between genome copies and infectious dose in cell culture (measured as tissue culture infectious dose 50% (TCID_50_) or, for some viruses that have a cytopathic effect (CPE), measured as plaque-forming unit, pfu; 1.0 TCID_50_ is approx. 0.69 pfu) are affected by several variables including the viral strain, the cell line chosen, the performance of the quantitative PCR and, importantly for coronaviruses, the choice of the amplified target within the genome; for, if the inoculum contains infected cells or cellular material, some quantitative RT-PCR assays will provide a very high RNA copies result because they detect not only the genomic RNAs but also the much more abundant nested mRNAs that are a hallmark of coronaviruses and other *Nidovirales*. In their review of published values, Sender *et al.* [16] reported a range of 10^3^–10^5^ genome copies per TCID_50_; more recently Hawks *et al.* [17] measured a ratio of approximately 200 genome copies per pfu.

## 3. SARS-CoV-2 in exhaled aerosols

It was noted previously that coughing and sneezing produce a large number of particles distributed over a wide size spectrum. It has also been established that activities such as singing, talking or even normal breathing produce a large number of aerosols and in fact mostly small aerosols [18–20]. If a liquid containing viruses in suspension is aerosolized, virions will predictably be encased in the resulting aerosol particles, and this has been shown in the laboratory (e.g. [21]). One would, therefore, predict that when infected with SARS-CoV-2, virus-containing aerosol particles would be found in air exhaled by patients. Of note is that in the generation of infectious bioaerosols, virions are typically encased in particles with a diameter much larger than the diameter of the virions; for SARS-CoV-2, virion diameters (without spikes) have a median of 0.1 µm, with spikes having a length of up to 0.023 µm [22].

Coleman *et al.* [23] have used a G-II exhaled-breath collector, which further separates collected aerosols into fine aerosols (less than or equal to 5 µm) and coarse aerosols (greater than 5 µm). Participants in Singapore were recruited based on molecular detection of SARS-CoV-2 and included symptomatic, asymptomatic and pre-symptomatic patients; collections were taken during breathing, talking and singing. In all, 59% of subjects emitted detectable levels of SARS-CoV-2 RNA, with loads ranging from 63 to 5821 N gene copies per expiratory activity; consistent with other observations [24], the detection was more likely in patients tested early in their infection. Of note, 85% of the RNA loads detected in the study were found in the fine aerosol collection. Attempts at isolation in cell culture were unsuccessful however.

Using the same methodology, Adenaiye *et al.* [25] reported the detection of SARS-CoV-2 RNA in 45% of fine aerosol samples and 31% of coarse aerosol samples in their subjects, with RNA levels ranging from 19 to 5.4 × 10^4^ copies per event. Very interestingly, infection with the alpha variant was associated with a 43-fold increase in fine aerosol viral RNA levels compared with earlier strains and variants not associated with increased transmission (there were no patients with the delta variant during the study). Isolation of SARS-CoV-2 in cell culture was successful with two fine aerosol samples with a load estimated at greater than 10^4^ RNA copies (one of which contained the alpha variant).

Such results are not unique to SARS-CoV-2; for example, using the same methodology with a G-II breath collector, Milton *et al.* [26] and Yan *et al.* [27] reported the presence of influenza A RNA in fine aerosols, with some samples yielding replicating viruses in cell culture; and Leung *et al.* [28] reported the detection by RT-PCR in fine aerosols of not only influenza A but also of rhinoviruses and human coronaviruses OC43, HKU1 and NL63. Using a SKC Biosampler, Lindsley *et al.* [29] demonstrated the presence of infectious influenza A viruses in aerosols (less than 10–15 µm) collected from cough and from exhaled breath in infected patients.

## 4. Laboratory studies on aerosolized SARS-CoV-2

Recovery of infectious viruses in cell cultures from aerosol samples is challenging. In the laboratory, however, aerosols with high viral loads can be created, which obviates the difficulties observed with low viral load samples; and considerable insight can be gained from measuring the half-life of the infectivity exponential decay.

Van Doremalen *et al.* [30] generated, from cultured virus stocks in cell culture medium, aerosolized suspensions of SARS-CoV-2 with diameters of less than 5 µm using a Collison nebulizer, and kept them suspended in a Goldberg drum at a RH of 65% and a temperature of 21–23°C. The authors demonstrated recovery of infectious viruses over several hours, with a measured half-life of 1.1–1.2 h. The RH chosen was not overly favourable to the survival of the viruses as aerosolized enveloped viruses typically remain infectious longer at lower RH.

Fears *et al.* [31] generated an aerosol suspension of SARS-Cov-2 in a similar manner and optimized parameters of the Goldberg drum to ensure a suspension of particles less than 2–3 µm, at a RH of 53% and at 23°C. They could recover infectious viruses for 16 h without observing a significant decay.

Smither *et al.* [32] used cultured SARS-CoV-2 to prepare aerosol suspensions of 1–3 µm particles using similar methods, comparing viral suspensions in either tissue culture medium or in artificial saliva after concentrating the virus stock using centrifugal filters, at temperatures of 19–22°C and at either medium RH (41–50%) or high RH (68–76%). Half-lives measured ranged from 30 min to 177 min, depending on the RH and the suspension medium.

Of note, SARS-CoV-1 and MERS-CoV have also been shown to remain infectious for hours in aerosols [30,31,33]. This is not unique to coronaviruses; for example, long survival in aerosols has been reported for influenza A virus [34] and measles virus [35].

## 5. Detection of SARS-CoV-2 in air samples

The logical inference from the previous sections is that if patients infected with SARS-CoV-2 produce aerosols containing viruses, including fine aerosols, and if aerosolized SARS-CoV-2 can maintain its infectivity for hours, then SARS-CoV-2 should be detectable in aerosol samples collected in the air of clinical environments.

Liu *et al.* [36] collected air samples at 30 sites in two designated hospitals in Wuhan dedicated to the treatment of COVID-19 patients, including intensive care units (ICUs), workstations, general wards, toilets and public areas. Air samples were collected with gelatin filters with a pore size of 3 µm; to obtain aerodynamic size segregation a Sioutas cascade impactor was used, enabling fractionation in five ranges (greater than 2.5 µm, 1.0–2.5 µm, 0.5–1.0 µm, 0.25–0.5 µm and less than 0.25 µm). In air samples collected without size segregation, about 53.3% of samples were positives with a load ranging from 1 to 19 RNA copies m^−3^. Measurements with size fractionation revealed segregation mostly in the 0.25–0.5 µm (peak concentration 40 RNA copies m^−3^) and greater than 2.5 µm (peak 9 copies m^−3^) fractions. No attempts at isolation in cell culture were made.

Guo *et al.* [37] collected air samples in ICUs and general wards in Wuhan where COVID-19 patients were hospitalized. They used a SASS 2300 Wetted Wall Cyclone sampler at 300 l min^−1^ × 30 min, which collects particles in the 0.5–10 µm range. In ICUs they collected samples in areas near air outlets, near patients and in doctors' offices area. SARS-CoV-2 was detected by RT-PCR. Overall, 37.5% of samples from air outlets were positive, 44.47% in patient rooms and 12.5% in doctors' offices. In general wards, the positivity rate of samples collected near patients was 18.2%, and 12.5% for air samples collected in the ward. No attempts at isolation in cell culture were made.

Chia *et al.* [38] conducted air sampling studies in Singapore by collecting aerosol samples with a NIOSH BC 251 sampler and detected SARS-CoV-2 by RT-PCR; samples were collected in three COVID-19 patient isolation rooms and were size-segregated into three ranges: less than 1 µm, 1–4 µm and greater than 4 µm. In 2 of 3 rooms, SARS-CoV-2 RNA was detected in the 1–4 µm fraction and greater than 4 µm fraction, with loads ranging from 916 to 2000 RNA copies m^−3^. No attempts at isolation in cell culture were made.

Santarpia *et al.* [39] collected air samples in COVID-19 patient rooms and hallways in a hospital in Nebraska, using a Sartorius Airport MD8 sampler at 50 l min^−1^ for 15 min; patients and staff were ambulatory, but there were no interactions of people with the sampler. Overall 63.2% of samples collected in rooms were positive for SARS-CoV-2 by RT-PCR, with a mean concentration of 2.42 copies l^−1^ of air (2.42 × 10^3^ copies m^−3^ of air); 58.2% of samples collected in hallways were positive with a mean concentration of 2.52 copies l^−1^ (2.52 × 10^3^ m^−3^ of air). The highest concentration was observed in the room of a patient receiving oxygen through a cannula, at 48.22 copies l^−1^ (4.82 × 10^4^ copies m^−3^). Although no size fractionation was done, detection in air samples from hallways indicates the presence of viral RNA transported by aerosols. As well, in a setting where the distance between people and the air collector was established to be at least 6 ft (1.83 m) at all times, 2 of 3 samples were positive for viral RNA. Attempts at isolation in cell culture, using Vero E6 cells, on a subset of positive samples did not yield a clear demonstration of infectious virus, although suggestive observations were made for two samples, including persistence or recurrence of RNA titre in the supernatant and virions observed by electron microscopy.

Nissen *et al.* [40] endeavoured to detect SARS-CoV-2 deposited in vent openings, air ducts and exhaust filters from COVID-19 patient rooms at distances and under a geometry that would preclude deposition from large droplets that followed ballistic trajectories, at the Uppsala University Hospital (Sweden). Detection of SARS-CoV-2 was done by RT-PCR and Sanger sequencing. The authors found that 36.8% of samples collected in vent openings were positive for SARS-CoV-2 RNA, as were 88.9% of samples collected from the main exhaust filters on the top floor of the hospital.

Fluid sample collections were also performed by placing open Petri dishes with tissue culture medium in the ventilation system, upstream of the exhaust filters. These showed a positivity rate of 33.3%. Attempts at isolation in Vero E6 cells were unsuccessful.

Lednicky *et al.* [41] conducted experiments with aerosol samples collected in the air of COVID-19 patient rooms. They used a VIVAS collector, with the collection process culminating with a water-based condensation of particles, thought to prevent damages to virions. The VIVAS collector has a 95% collection efficiency in the 0.008–10 µm range [42]. Some samples were collected with a commercial implementation of the VIVAS instrument. SARS-CoV-2 was detected by RT-PCR in 4 of 6 samples (2/3 for each bedroom sampled), with estimated RNA loads ranging from 16 to 94 RNA copies l^−1^ (1.6 × 10^4^–9.4 × 10^4^ copies m^−3^). Isolation in cell culture, using Vero E6 cells and LLC-MK2 cells, was actually successful in both cell lines for all these four positive samples, as demonstrated by the observation of CPE within 4–6 days post inoculation (DPI), positive RT-PCR on culture supernatant at several DPIs, with negative detection of other respiratory viruses as demonstrated by the use of the Biofire Film Array Respiratory 2 Panel (BioMerieux). An attempt was also made by the authors to establish the infectious titre in air samples using calibration curves between RNA copies and TCID_50_, but the ratio of TCID_50_ to genome copies appears higher than that found by other authors.

In Ledniky *et al.* [43], the authors collected aerosol samples in the air of a car driven by a mildly symptomatic (but not coughing) patient with a laboratory-confirmed SARS-CoV-2 infection. The patient drove the car for a short period of time while the collection was ongoing, and the collection continued on the residual air for a total collection time of 135 min allowing for a sampling of about 1.22 m^3^. The authors used a Sioutas Personal Cascade impactor sampler clipped onto the sun-visor above the passenger seat, which would not be accessible to large droplets following a ballistic trajectory from the driver seat. Furthermore, the Sioutas collector allows for size segregation, in five size fractions of greater than 2.5 µm, 1.0–2.5 µm, 0.5–1.0 µm, 0.25–0.5 µm and less than 0.25 µm. Recovery from the sampler occurred within 30 min after the end of the collection. Detection of SARS-CoV-2 by RT-PCR was positive in 4 of 5 fractions (no detection in the less than 0.25 µm fraction), with estimated RNA loads ranging from 1.24 × 10^3^ to 3.14 × 10^4^ RNA copies m^−3^ of air. Isolation in Vero E6 cells was successful using the 0.25–0.5 µm fraction (which contained the highest amount of RNA copies), as demonstrated by CPE and detection in the supernatant of SARS-CoV-2 by RT-PCR and sequencing.

Finally, Santarpia *et al.* [44] also demonstrated the presence of infectious SARS-CoV-2 in aerosol samples. Using a NIOSH BC25 sampler the authors collected aerosols samples in five patient rooms in two different hospital wards; the samples were size-segregated in three groups: greater than 4.1 µm, 1–4 µm and less than 1 µm. A gelatin filter was used in the final stage to minimize damages to the virions.

Detection of SARS-CoV-2 by RT-PCR was demonstrated in samples from all size groups. Isolation of SARS-CoV-2 in Vero E6 cells was attempted with all 18 positive samples; in five samples (two in the 1–4 µm group and three in the less than 1 µm group), the presence of infectious viruses could be demonstrated by increased viral RNA in the culture supernatant, using quantitative RT-PCR, over several DPIs (statistical significancy was reached for the samples in the 1–4 µm group); this was corroborated by western blot analysis showing the synthesis of nucleocapsid proteins. Transmission electron microscopy showed the presence of coronavirus virions.

## 6. Outbreaks involving aerosol transmission

Studies reviewed in the last section established that aerosolized SARS-CoV-2 is reproducibly detected in the indoor environment of infected patients, and infectivity in cell culture has been documented in some cases which, given fundamental limitations, are almost certainly an underestimate.

Yet all these studies also showed a rather low average concentration in the air. But this is actually a common finding for many aerosol-transmitted infections. For example, in their seminal study on airborne transmission of tuberculosis, Riley *et al.* [45] established that on their tuberculosis ward the average concentration was about 1 bacillus per 12 500 to 11 000 cubic ft (approx. 2.83 × 10^−3^–3.21 × 10^−3^ bacilli m^−3^).

This apparent paradox is resolved in part by considering the large volume of air inhaled over 24 h by an average person (at an average minute volume of 6 l min^−1^ this would translate into about 8.6 m^3^ per 24 h [20]); this creates the possibility of an accumulated dose reaching the threshold of an infectious dose. As well, the average air concentration of a pathogen is just that—an average. The concentration of aerosols is not uniform in a room and indeed would be significantly greater in the proximity of an infected patient; in indoor environments, ventilation and air currents are often not homogeneous and create uneven stirring. Also, and even with homogeneous stirring, aerosols will disperse randomly which is not the same thing as a uniform distribution [46]; this is a consequence of having a small number of particles dispersing in a large volume, resulting in infectious particles separated by large and uneven volume of non-infectious air [46] (if the number of particles would be much larger, as with a gas, then the random distribution would converge to a uniform distribution). Furthermore, patients are very heterogeneous in their shedding of viruses, including the natural temporal variation during infection with a peak from just prior to symptoms onset to a few days afterward [24], the difference between individuals in their generation of bioaerosols [18] and the finding that some patients are considerably more contagious than average and are known as ‘super-spreaders’; the occurrence of super-spreaders has been well documented for SARS-CoV-1 [47] and for SARS-CoV-2 [48].

Lastly, aerosol-transmitted diseases would typically have a low infectious dose, at least with an aerosol inoculum: for example, the human infectious dose 50% (HID_50_) of influenza A virus is much smaller by aerosols than by nasal instillation (reviewed in [49]). At this time there is no good measurement of the HID_50_ of SARS-CoV-2, and given the virulence of SARS-CoV-2, direct laboratory measurements with volunteers would be ill advised. It is also becoming understood that the mutations required for SARS-CoV-2 to grow well in Vero and related cell lines are not the same than those required for growth in primary differentiated human bronchial epithelial cells [50], so that the ratio of virions to TCID_50_ may not be an accurate measurement of the ratio of virions to HID_50_.

Be that as it may, there is a way to establish empirically that aerosol transmission of SARS-CoV-2 occurs by examining reported outbreaks which strongly point toward aerosol transmission, or indeed where it is an inescapable conclusion.

Shen *et al.* [51] reported early in the pandemic an outbreak among bus passengers in eastern China, within Nigbo City at a time where there had been no confirmed cases of COVID-19. The bus was one of two buses used by a party attending an event that included luncheon with random assignation of seats. By contrast, passengers had an assigned seat in buses and did not change seats, even on the return trip, or moved around in the bus. Both buses had an air-conditioned system that operated in recirculated air mode, which is propitious for an increase of the aerosol concentration over time. All cases were linked to an index case who was the only person exposed to residents from Wuhan prior to the journey; 23 primary cases were among the 60 riders in the bus with the index case. There were no cases in the other bus. Seven other attendees at the event were subsequently diagnosed with COVID-19 (all of whom reported close contacts with the index), as well as the child and spouse of the index case. The spatial distribution of the 23 cases in the bus showed a random scattering, with some passengers next to the index case having remained uninfected while other infected cases were seated several rows away. As noted above, a basic property of aerosols is that they will follow a random distribution within an enclosed space, which is not the same thing as a uniform distribution [46]. Careful history taking along with the lack of other cases in the community at the time provides a high degree of certainty to the transmission chain. A commonly voiced criticism of this study is that several cases occurred after an alleged suspiciously long incubation time. In fact, 25 of the 32 cases occurred within 13 days and only two cases occurred beyond day 17. The 95% percentile for incubation time has been estimated at 12.5 days [52]; furthermore, incubation time increases with age and the 90% percentile for older adults has been estimated at 17 days [53], all of which does not make this criticism overwhelmingly convincing.

An outbreak involving three family clusters in a restaurant in Wuhan has been initially described by Lu *et al.* [54] and analysed in depth by Li *et al.* [55]; it involved a total of 10 cases distributed along three tables in a row with the index case sitting in the middle table. Although some of the cases would have occurred through subsequent intra-family infections, initial infections in each cluster must have occurred at the restaurant at a time when there had been only 322 cases among the 13 million inhabitants of Wuhan. Infections between the tables would have involved distance greater than 1 m and up to 4.6 m [55]. None of the other adjacent tables and indeed no other persons in the restaurant at that time, including waiters, became infected. An extensive study of the air flow generated by thermal plumes and wall-mounted air conditioning units in recirculating mode, involving the use of smoke tracers and computational simulations, showed that all three tables were within an air recirculation bubble created by the air conditioning units at a time when exhaust fans were not in operation, with a resulting ventilation rate of no more than 1.04 l s^−1^ per person. It is striking that all cases occurred within that bubble, none occurred outside. An initial review of the security camera video recording failed to reveal opportunities for fomite or close contact transmission. A follow-up study [56] provided an in-depth review of video recordings in the restaurant, from three high-resolution cameras over a 2 h 20 min period. In all, 40 935 surface touches and more than 13 000 episodes of close contacts were recorded. Analysis of possible fomite close contacts under eight different combinations of index patient failed to reveal a significant correlate between the estimated relative risk and the reported infection data.

Hamner *et al.* [57] initially reported on a super-spreading event at a choir practice in Skagit County (Washington, USA). The choir included 122 members; it held several practices during the month of March. Identification of an index case, through symptoms occurring a few days before the event and confirmed by laboratory testing, led to an in-depth investigation pinpointing the 10 March practice as the very likely point-source exposure event. Among the 61 persons who attended the practice, there were 32 confirmed and 22 probable cases that led to 3 hospitalizations and 2 fatalities. The temporal pattern of symptoms onset is consistent with a point-source outbreak, at a time where there was no known COVID-19 case in the county [58]. A careful review allows for a possible role in some cases for large droplets or fomites although precautions were observed by the participants including the use of hand sanitizer, and a proscription of handshakes or hugs; indeed no physical contact was reported by the participants upon questioning. Chairs were about 0.75 m apart in rows, with a 1.4 m distance between rows. Little is known about the effective ventilation during the practice; doors and windows were closed. The heating system of the hall was a forced-air system that included a MERV-11 filter (these have a 65% filtrating efficiency in the 1–3 µm range), but it is likely that it would not have run for the whole evening given the outside temperature at the time [58]. Given the magnitude of the attack rate, it appears unlikely that the whole outbreak can be explained solely by large droplets or fomites. Loud singing (or loud speaking for that matter) is associated with a significant increase in bio-aerosol emission [59]. A modelling of the outbreak, assuming aerosol transmission following the Wells–Riley formulation was performed using Monte Carlo simulations with different values for infectious quanta emission rate during singing, and yielded infection probabilities in keeping with the observed number of cases [58].

Azimi *et al.* [60] established a model of the outbreak on the Diamond Princess cruise ship. The outbreak started with a single infected passenger, and resulted in 712 cases among passengers and crew members, with an additional 57 cases that tested positive days after their departure from the ship. The outbreak initial phase of transmission was followed by a period of quarantine where all passengers were confined to their cabins. Modelling benefitted from a wealth of data including a high degree of knowledge of the human and built environment factors, high rate of testing and high number of cases over time. The approach of the authors involved Markov chain models, with the modes of transmission considered being long-range aerosol transmission, short-range aerosol transmission, short-range large droplets and transmission through direct contacts/fomites. Back calculations for HID_50_ were done using the first 5 days of the simulation period as a calibration period, and a range of parameter values was used in several different simulations. Overall, in all simulations under all hypotheses, transmission by small aerosols (less than 10 µm) through both long-range and short-range transmission constituted the most likely dominant mode, accounting for more than 50% of all cases. This was so even though the model and simulations assumed a high ventilation rate with no air recirculation. The authors used the cut-off value of less than 10 µm but acknowledged that emitted particles from the respiratory tract are on a size continuum, and pointed out that if aerosols had been defined as less than 100 µm the aerosol contribution to the outbreak would have been shown to be even greater.

Katelaris *et al.* [61] reported evidence for aerosol transmission involving 12 secondary cases identified following a contact investigation of a church chorist diagnosed with COVID-19. Investigation of all church attendees during what would have been a period of high contagiosity identified 12 cases over a period of 2 days. Full-length genomic sequencing of all the isolates in these cases showed that they were all part of a genomic cluster with no more than 2 nt differences. The chorist entered in the church on an elevated (3.5 m) choir loft through a side door and sat at a piano, with his back turned to the audience. Horizontal distances between the singer and cases ranged from 5 m to 15 m. Site inspection revealed that almost all doors and windows were closed and that ventilation fans were off. History taking established a lack of direct contact between the index cases and the chorist, corroborated by security video recordings. Given the distance and geometry involved in this outbreak, transmission by large droplets would have been clearly impossible.

Günther *et al.* [62] provided an in-depth investigation of a very large outbreak at a meat processing plant in Germany. There have been several reports worldwide of significant outbreaks in meat processing plants, for which factors such as proximity of workers in assembly lines, shared transportation and even shared housings have been invoked as contributory. However, the hard physical work and the necessity of shouting because of ambient noise create opportunities for an increased bioaerosol generation; the cold and dry air maintained in such plants favour infectivity persistence of the aerosolized viruses, and air is typically recirculated for energy conservation while providing refrigeration; all this points to a very plausible aerosol contribution to these outbreaks. In the plant under consideration, wall-mounted air conditioning units in effect sectioned the meat processing room into zones of recirculated air [62]. An initial outbreak in May involved 94 cases; full genomic sequencing of 20 cases showed that all the cases were caused by a hitherto undescribed sub-branch within the 20C clade. Of the two probable index cases, one had an isolate with an additional mutation not found in any other case and was ruled out as an index case. Analysis of cases among workers with a fixed working position showed a statistically significant over-representation of being in a radius of 8 m from the index case; no other significant correlation was found looking at shared carpools, shared apartments or bedrooms. The authors concluded that since many transmission events occurred within the plant at distances of up to 8 m, aerosol transmission played a significant role. A subsequent larger outbreak in June involved more than 1400 cases and was probably seeded by, or a continuation of, the first outbreak since 15 samples taken early in the outbreak showed that all the isolates belonged to the same subclade albeit with the introduction of minor additional mutations.

Wearing a surgical or procedural mask has long been advocated as an effective way to decrease transmission of a pandemic respiratory agent within the population (e.g. [63]). The filtrating efficiency of surgical masks varies between brands but can achieve high level of filtration down to a few micrometres [64]. In addition to conferring protection to the wearer, wearing a mask significantly decreases large droplet and aerosol particle emission, including for micrometre size particles [25,65,66]. The beneficial impact at a population level of mask wearing on SARS-CoV-2 transmission has been verified (e.g. [67,68]). However, if aerosol transmission occurs at a significant level there ought to be instances where the transmission will occur in spite of greater than 2 m distanciation and wearing of surgical masks, through either penetration of small aerosols through the mask or aerosols following an air current around loose side opening (in contrast to surgical masks, respirators such as N95s or FFP2s create a hermetic seal around the airways). Such instances of SARS-CoV-2 transmission have in fact been documented in the literature. Klompas *et al.* [69] reported three instances of transmission in the healthcare setting, in scenarios of mask worn by the source, mask worn by the recipient and masks worn by both. Transmissions were verified by the identity of the viral genomic sequences. Goldberg *et al.* [70] described a single-source nosocomial outbreak in which a single patient transmitted SARS-CoV-2 to six healthcare workers (HCWs) and three other patients; all the HCWs wore surgical masks and eye protection; three of the HCWs had no direct contact and maintained a distance of at least 6 ft (1.83 m).

## 7. Animal models

SARS-CoV-2 has a rather broad species tropism, which includes laboratory animals. Development of animal models allows for highly controlled experiments and for disentanglement of aerosol, large droplet and contact/fomites modes of transmission; insight about pathogenesis can also be gained, although species differences must be kept in mind.

Studies have naturally been done on non-human primates. Hartman *et al.* [71] have reported that African green monkeys (AGMs) can be successfully infected with SARS-CoV-2 by instillation done simultaneously at multiple mucosal sites (nares, oral and ocular mucosa, trachea) with a total dose of approximately 2.5 × 10^6^ pfu. The authors could also demonstrate infection by exposure to an aerosol inoculum obtained with an Aerogen Solo nebulizer, with a mass median aerodynamic diameter of 1.7 µm. The calculated inhaled doses ranged from 10^3.7^ to 10^4.2^ pfu. All the infected animals shed viruses in respiratory mucosa and seroconverted, but had only a mild clinical illness although PET scan imaging revealed lung lesions that resolved over time.

Johnston *et al.* [72] also reported successful infection of AGMs by the aerosol route; the inocula, generated using a Collison nebulizer, had a mass median aerodynamic diameter of 1–3 µm and the calculated mean inoculated dose was 3.8 × 10^4^ pfu; clinical illness was mild but did include mild hypoxia and dyspnea. Johnston *et al.* [72] also reported successful infection by aerosols of rhesus macaques, who remained only mildly symptomatic, and cynomolgous macaques (CM) which were infected with an average dose of 4.86 × 10^4^ pfu; in contrast to the observations of Rockx *et al.* [73] on CMs infected by mucosal inoculation with a combined intratracheal and intranasal instillation with a total dose of 10^6^ TCID_50_, and who remained asymptomatic, the CMs infected by the aerosol route displayed clinical illness and, significantly, had fever. This difference in the clinical presentation of CMs depending on the route of transmission suggests that SARS-COV-2 displays *anisotropy*. Anisotropic infection is a concept formally introduced by Milton [74] for some infectious agents that can be transmitted by different routes of transmission with different effectiveness and/or severity of the resulting illness; thus for smallpox the mortality associated with aerosol infection is significantly greater than by subcutaneous inoculation (variolation). Influenza A also displays anisotropy, as discussed in [49].

Port *et al.* [75] reported that Syrian hamsters can be experimentally infected by SARS-CoV-2 by exposure to either contaminated fomites (8 × 10^4^ TCID_50_), nasal instillation (8 × 10^4^ TCID_50_ in a 40 µl volume) or aerosol (1.5 × 10^3^ TCID_50_) administered over 10 min with an aerosolized inoculum prepared with a Collison nebulizer, with diameters ranging from 1 to 5 µm. The authors showed that compared to infection through fomites, infection acquired by either nasal instillation or aerosols resulted in a significantly more severe disease with significant weight loss; thus the hamster model also displays anisotropy. The increased disease severity associated with aerosols for anisotropic agents is thought to be in great part attributable to direct access of the inoculum to the lower respiratory tract. Indeed Port *et al.* have shown that in all animals infected by aerosols there were viruses deposited in both the upper and lower respiratory tract, with infectious viruses detected in the lungs at 1 DPI. Avoiding immediate deposition in the lower respiratory tract with intranasal instillation can be technically challenging. For example in a mouse model for influenza A virus, Larson *et al.* [76] showed that nasal instillation using a 50 µl volume invariably resulted in pulmonary aspiration and was not, therefore, an inoculum restricted to the upper respiratory tract; aspiration was no longer observed with inoculum volume of 1 µl [76]. It is not clear whether aspiration can occur using a 40 µl instillation volume in a Syrian hamster (which is after all a larger animal) although Port *et al.* noted that infectious viruses in the lungs at 1 DPI was observed in a fraction of animals inoculated by nasal instillation; this is a point that may warrant further elucidation.

Zhang *et al.* [77] demonstrated that CMs experimentally infected with SARS-CoV-2 exhaled aerosol-size particles containing SARS-CoV-2, with peak shedding occurring at 2 DPI; the average exhaled load was 106 viral RNA-containing particles min^−1^; the size distribution was measured and revealed a distribution of 27.4%, 49.6% and 23.0% in the size fractions 0.65–2.1 µm, 2.1–4.7 µm and greater than 4.7 µm, respectively. Attempts at isolation in cell culture were unsuccessful.

Hawks *et al.* [17] also showed that experimentally infected Syrian hamsters exhaled aerosol-size particles containing viral RNA, at a rate of 700 particles min^−1^ with 99.9% of the particles in the size fraction less than 10 µm. Air samples were also collected with a condensation sampler to preserve infectivity. The presence of infectious viruses was demonstrated by isolation in Vero cells, with a mean emission rate of 1.4 log_10_ pfu h^−1^ over the first 2 DPI; additional air samples were collected from the air chamber using a cyclone separator. The authors could demonstrate recovery of infectious virus in the size fraction less than 5 µm.

The demonstration of exhaled aerosolized SAR-CoV-2 in experimentally infected animals raises the question of whether aerosol transmission between animals can be accomplished in the laboratory. Kutter *et al.* [78] used the ferret experimental model with a setting involving an experimentally infected ‘donor’ animal in the bottom cage and a ‘recipient’ ferret in a top cage connected to the bottom cage with a duct system involving four right angles turns and a 118 cm vertical section. Given the dimensions of the duct and the airflow rate stipulated in the text, one can ascertain the air speed in the vertical section and, applying Stokes' Law [2], determine that the maximum aerodynamic diameter of particles exchanged would be about 56 µm. With this set-up, Kutter *et al.* [78] could demonstrate the successful aerosol transmission of influenza A(H1N1), SARS-CoV-1 and SARS-CoV-2.

Finally, Port *et al.* [79] used the Syrian hamster model in an experimental set-up involving two cages connected in such a way that only particles less than 10 µm in diameter could be transferred between cages and in fact most particles were in the less than 5 µm range (physical diameter, as measured with aerosolized glycerin 20% solution). The authors could demonstrate the efficient aerosol transmission of SARS-CoV-2 as established by sero-conversion and virus shedding in the recipient animals. Furthermore, competition between viruses belonging to the original lineage and to the lineage B.1.1.7 (alpha variant) demonstrated the greater capacity of the alpha variant to infect by the aerosol route.

## 8. Summary and discussion

This review makes the case for aerosol transmission of COVID-19 based on several different lines of evidence. It starts with basic respiratory physiology and how coughing and sneezing, and also other respiratory activities such as singing, talking and just breathing, generate aerosol-sized particles from the epithelial lining fluid. Laboratory data confirm the expectation that aerosolized liquid suspensions of viruses result in aerosols containing virions. It would then be expected that patients infected with SARS-CoV-2 would exhale aerosol-size particles containing virions. This has now been established experimentally, not only by molecular detection of the viruses in aerosol samples but also by isolation in cell culture, which establishes the infectivity of aerosolized viruses. It is important to note that isolation in cell culture is significantly less sensitive than detection by molecular methods and consequently detection by culture methods underestimates aerosol infectivity.

It is followed by the review of laboratory investigations showing that aerosolized SARS-CoV-2 (and other coronaviruses) remain infectious for typically hours.

The next link in the chain is the detection of SARS-CoV-2-containing aerosols in the air of indoor environments where infected patients are present; most studies reviewed involved hospitals but one important example involved air samples from a passenger car. Viruses were detected by molecular methods, and in some cases also by isolation in cell culture; great attention was given to the size cut-off for the aerosol samples.

Outbreak investigations play an important role as they can in principle provide empirical validation that aerosol transmission occurs and in fact occurs extensively. Substantiation of aerosol transmission in outbreak investigations required that great attention be given to distinguishing properties of aerosols; for example, even though short-range transmission of aerosols would occur, it could not be easily disentangled from transmission by large droplets or direct contacts. Long-range transmission, if well established, essentially proves aerosol transmission; but it is expected to occur at significant levels when ventilation is poor or defective—which was a hallmark of almost all studies presented (which also often involved superspreading events). Great care must be taken to establish that infections of patients did take place within the outbreak; this can be done in a variety of ways, for example, if the outbreak occurred at a time where there was very little community transmission, or when patients were confined to a cruise ship, or if there was a genetic marker unique to the virus strain implicated in the outbreak. Ruling out as much as possible transmission by other routes during the outbreak required documentation of lack of direct contact or of close proximity through the use of questionnaires, corroboration by security camera recordings, or statistical analysis. All the outbreaks reviewed point to a significant role for aerosol transmission and, in some cases, it appears to be an inescapable conclusion.

Experimental animal models for SARS-CoV-2 infection allow for a completely controlled environment which allows for the disentanglement of the different modes of transmission. Experimental animals could be infected experimentally through different routes, including by aerosol inocula—and there were indications of an anisotropic effect. Infected animals exhaled aerosolized infectious viruses, and animal to animal transmission by aerosols was demonstrated.

Lastly, whereas almost all the studies reviewed here involved SARS-CoV-2 strains other than variants of concern (currently alpha, beta, gamma and delta), some evidence has been presented that the more contagious and more virulent alpha variant is also more proficient at aerosol transmission. Studies on the delta variant are eagerly awaited.

This review has benefited from several studies only recently published, but many other reviews and commentaries published earlier in this pandemic had also arrived at the conclusion that aerosol transmission plays a very important role (e.g. [8,9,80–84]).

Pending the completion of the global vaccination endeavour, a comprehensive programme for interruption of SARS-CoV-2 must include control of aerosol transmission. Whereas surgical mask wearing has a documented beneficial effect at a population level, higher grade PPE (N95 or better) are clearly required in some settings including when providing care to COVID-19 patients. Healthcare workers have been disproportionally infected during the pandemic [85]. Since a basic property of aerosols is that they are removed by ventilation [46] or, putting it another way, that aerosols will linger and get concentrated in the vicinity of infected patients in the absence of adequate ventilation, sufficient ventilation is, therefore, a key countermeasure, which can be also supplemented by air filtration when appropriate [86].

There are now large amounts of data supporting the contention that viruses with proven pandemic capacity within two virus families, *Orthomyxoviridae* and *Coronaviridae*, are efficiently transmitted by aerosols. This is not the last pandemic we will have to face, and no doubt some of them will involve emerging viruses capable of aerosol transmission. It is, therefore, imperative that infection control protocols be updated, and adequate stockpiles of PPE and adequate manufacturing capacity be assured; as well, our current infrastructure, building construction and management of indoor spaces must be re-evaluated to provide a higher degree of aerosol protection [87].

## Data accessibility

All references are available as scientific publications.
