# Correction to ‘COVID-19: the case for aerosol transmission’ 2022 by Tellier

**DOI:** 10.1098/rsfs.2022.0060

**Published:** 2022-12-09

**Authors:** Raymond Tellier

*Interface Focus* **12**, 20210072. (Published 11 February 2022). (https://doi.org/10.1098/rsfs.2021.0072)

Please note the following correction. In §3 (page 2, right-hand column), the sentence 'Isolation of SARS-CoV-2 in cell culture was successful with two fine aerosol samples with a load estimated at greater than 10^4^ RNA copies (one of which contained the alpha variant).' should read as follows:

Isolation of SARS-CoV-2 in cell culture was successful with two fine aerosol samples, one of which contained the alpha variant with a load estimated at greater than 10^4^ RNA copies.

